# Electrochemotherapy combined with immunotherapy – a promising potential in the treatment of cancer

**DOI:** 10.3389/fimmu.2023.1336866

**Published:** 2024-01-15

**Authors:** Benjamin Hadzialjevic, Masa Omerzel, Blaz Trotovsek, Maja Cemazar, Tanja Jesenko, Gregor Sersa, Mihajlo Djokic

**Affiliations:** ^1^ Department of Abdominal Surgery, University Medical Center Ljubljana, Ljubljana, Slovenia; ^2^ Department of Surgery, Faculty of Medicine, University of Ljubljana, Ljubljana, Slovenia; ^3^ Department of Experimental Oncology, Institute of Oncology Ljubljana, Ljubljana, Slovenia; ^4^ Faculty of Health Sciences, University of Primorska, Izola, Slovenia; ^5^ Faculty of Health Sciences, University of Ljubljana, Ljubljana, Slovenia

**Keywords:** cancer, electrochemotherapy, electroporation, immunotherapy, immune response, melanoma, breast cancer, hepatocellular cancer

## Abstract

Electrochemotherapy is a novel, locoregional therapy that is used to treat cutaneous and deep-seated tumors. The electric pulses used in electrochemotherapy increase the permeability of the cell membranes of the target lesion and thus enhance the delivery of low-permeant cytotoxic drugs to the cells, leading to their death. It has also been postulated that electrochemotherapy acts as an *in situ* vaccination by inducing immunogenic cell death. This in turn leads to an enhanced systemic antitumor response, which could be further exploited by immunotherapy. However, only a few clinical studies have investigated the role of combined treatment in patients with melanoma, breast cancer, hepatocellular carcinoma, and cutaneous squamous cell carcinoma. In this review, we therefore aim to review the published preclinical evidence on combined treatment and to review clinical studies that have investigated the combined role of electrochemotherapy and immunotherapy.

## 1 Introduction

Electrochemotherapy is an emerging, locoregional ablative therapy that uses electric pulses to increase the permeability of cell membranes to facilitate the entry of chemotherapeutic agents into cells. The most commonly used chemotherapeutic agents are bleomycin and cisplatin (1). The first clinical applications of electrochemotherapy were published in the 1990s, and with the accumulation of results from multiple studies, standard operating procedures (SOPs) were published in 2006, followed by an updated SOP for electrochemotherapy in 2018 (2, 3). Indeed, electrochemotherapy is nowadays predominantly used in the treatment of cutaneous tumors, however, several studies have been published to date on the treatment of deep-seated tumors (4–7).

In addition to electrochemotherapy, other ablation therapies are also widely used to treat various cutaneous and deep-seated malignant and benign tumors. The most common ablation therapies include cryoablation, radiofrequency ablation (RFA), microwave ablation (MWA), irreversible electroporation and electrochemotherapy as mentioned earlier (1, 8). Initially, the response to ablation therapies was attributed only to local elimination of tumor by various mechanisms of chemical and physical effects. Recently, however, it has been recognized that ablation promotes tumor antigen release, increases tumor antigenicity and accordingly triggers a systemic antitumor immune response that can then be fully exploited in combination with immune-checkpoint inhibitors (ICIs) (9).

Immunotherapy with ICIs has revolutionized cancer treatment, providing patients with significant improvements in survival and quality of life (10). Essentially, immune checkpoints (the best known are CTLA-4 and PD-1) are receptors expressed on the surface of T-cells and are responsible for negatively regulating the T-cell mediated immune response during the cancer immune-editing process. Accordingly, ICIs suppress these receptors in order to reactivate the immune response against tumor cells (11). ICIs are now approved for the treatment of numerous cancer types, including advanced melanoma, lung cancer, head and neck squamous cell carcinoma, hepatocellular carcinoma (HCC), Merkel cell carcinoma, urothelial and renal carcinoma, Hodgkin’s lymphoma, triple-negative breast cancer, cervical and endometrial carcinoma, and gastric cancer. Although ICIs are generally very effective, a relatively high percentage of patients still do not respond to treatment (11, 12). Thus, ongoing research into new treatment strategies is mandated to address this problem.

In this review article, we aim to discuss electroporation and electrochemotherapy in general, evaluate the mechanisms of the synergistic effect of combined electrochemotherapy and immunotherapy, and review the published clinical applications of combined treatments.

## 2 Electrochemotherapy – mechanisms of action

Electrochemotherapy is now used throughout Europe, particularly for skin tumors of various histologies. The reason for its relatively rapid translation from bench to bedside was its efficacy and, more importantly, in the early stages, the known mechanisms of action (13). The application of electric pulses in the kV range with a duration of microseconds leads to structural changes in the cell membrane that enable the transport of molecules in and out of the cells. Immediately after the application of the electric pulses to the target tissue, the changes begin to close or return to their previous organization. The application of such pulses is used in so-called reversible electroporation for the administration of chemicals, e.g. in electrochemotherapy with bleomycin or cisplatin, calcium electroporation, where the main goal of the therapies is cell death, and for the administration of nucleic acids in gene electrotransfer, where the goal is to obtain expression of therapeutic transgene. When multiple electric pulses are delivered to the tissue, this can lead to irreversible electroporation, in which severe disruption of the cell membrane leads to irreparable damage to the cell and thus to cell death, without the need for a cytotoxic drug as in electrochemotherapy (14). The drugs used in electrochemotherapy are bleomycin and cisplatin, which are hydrophilic drugs with hindered transport across the cell membrane. Therefore, electroporation of the cell membrane, i.e. the application of electric pulses, enables the transport of these two cytotoxic drugs into the cells (13). The effectiveness of cytostatics, which are widely used in the treatment of cancer, is therefore only enhanced by introducing more cytostatics into the cells of the tumors. The effect is only present in the tissue in which the electric pulses were delivered. The enhanced effect of cytostatic drugs is therefore limited to the treated area. As efficacy is potentiated in this way, the drug concentration applied is low and does not cause any systemic or local undesirable side effects (15).

As noted, the main mechanism of electrochemotherapy is the delivery of cytotoxic drugs to tumor cells exposed to the electric field (13). In addition to the cytotoxic effect on the tumor cells, electrochemotherapy also affects tumor blood vessels by electric pulses alone and the delivery of the drug to the tumor vasculature, especially to the endothelial cells (16). Essentially, the application of electric pulses leads to an immediate vasoconstriction of the tumor vessels (i.e. vascular lock effect) that lasts for several hours. This in turn is followed by a delayed vascular disrupting effect (17). The cumulative effect on tumor blood vessels is further enhanced by the addition of bleomycin (18). Electroporation also increases the permeability of the affected endothelial cells which in turn take up bleomycin and eventually die when they begin to divide. Since endothelial cells divide rapidly, the vascular disruptive effect of electrochemotherapy is rapid and the destruction of the tumor vasculature resulting in a permanent reduction in blood flow to the tumor becomes pronounced on the order of hours and days. Moreover, it has been shown that electrochemotherapy has a selective vascular disrupting effect on tumor vasculature, without affecting normal blood vessels which surround the tumor (18–20).

The third, very important mechanism is the induction of the immune response by inducing immunogenic tumor cell death, which can trigger the local immune response (21). The first studies have shown that some immune response is induced after electrochemotherapy of tumors in mice (22). The study on sarcoma-bearing mice then tested whether the tumor response depends on the presence of the immune response. The study showed that the tumor response was lower in immunodeficient mice and, more importantly, that the absence of the immune response abrogated the complete tumor response. This suggests that an immune response must be present for complete eradication of tumors and that electrochemotherapy itself must play some role in eliciting the response (23). In addition, a comparison of tumor responses according to differences in tumor immunogenicity showed that more immunogenic tumors responded better than less immunogenic tumors (24).

As the importance of immunogenic cell death in cancer therapy has been recognized (25), the role of electrochemotherapy as well as other ablative therapies in eliciting immunogenic cell death leading to a local and systemic immune response has been investigated (26). Various ablative therapies such as ionizing radiation, RFA, cryotherapy, and irreversible electroporation, have been found to induce a local immune response by triggering immunogenic cell death (27). This has also prompted researchers to investigate immunogenic cell death by electroporation itself and specifically by electrochemotherapy (28, 29). Essentially, immunogenic cell death is characterized by the release of damage-associated molecular patterns molecules (DAMPs). These molecules are normally present intracellularly and cannot penetrate cell membranes. However, when they are released from cells (e.g. in trauma or other states of increased cell permeability), they can boost innate and adaptive immune response by activating other cells involved in the immune response (30). In addition, other cytokines, chemokines and inflammatory markers are often released along with DAMPs (31). In the early studies on electroporation, DAMPs (especially ATP) were used as indicators of successful permeabilization of the cell membrane (32). Therefore, in a recent study, Polajzer et al. investigated if and when specific DAMPs (i.e. ATP, calreticulin, nucleic acids and uric acids) are released as a consequence of electroporation itself. They showed that the release of DAMPs increased with increasing pulse amplitude, while the concentration of most DAMPs correlated strongly with cell death, suggesting that DAMPs may serve as markers for the prediction of cell death (28).

In our study by Ursic et al. (29), we found that several parameters indicated that the immune response was triggered by electrochemotherapy with either bleomycin, cisplatin or carboplatin. It was found that there were differences in the activation of the immune response between the tumors and the drugs used for electrochemotherapy. In particular, electrochemotherapy was more effective when the tumors were more immunogenic (CT 26 murine colorectal carcinoma) than when they were less immunogenic (4T1 murine mammary carcinoma and B16F10 murine melanoma). In continuation of this study, we supported these data by an *in vitro* assessment of immunologically important changes in tumor cells after electrochemotherapy with different inhibitory concentrations of the drugs leading to different degrees of cell death. We evaluated in murine tumor cell lines B16F10, 4T1, and CT26 whether electrochemotherapy triggered changes in immunogenic cell death DAMPs: calreticulin, ATP, high mobility group box 1 (HMGB1), and four immunologically important cellular markers: MHCI, MHC II, PD-L1 and CD40. Again, similar to the *in vivo* results, electrochemotherapy with all three chemotherapeutic agents tested induced DAMPs, but the induced DAMP signature was cell line and chemotherapeutic concentration specific. Similarly, electrochemotherapy altered the expression of MHC I, MHC II, PD-L1 and CD40, which was also cell line and chemotherapeutic concentration specific (33). The data obtained from *in vitro* and *in vivo* studies have shown that electrochemotherapy indeed induces immunogenic cell death and is therefore a suitable candidate for combination with ICIs (34). Nevertheless, further studies in this direction are needed, as understanding the biological factors that influence tumor response to electrochemotherapy will enable better treatment planning and combination with immunotherapy (35).

## 3 Intersection of electrochemotherapy and immunotherapy

**Figure 1:**
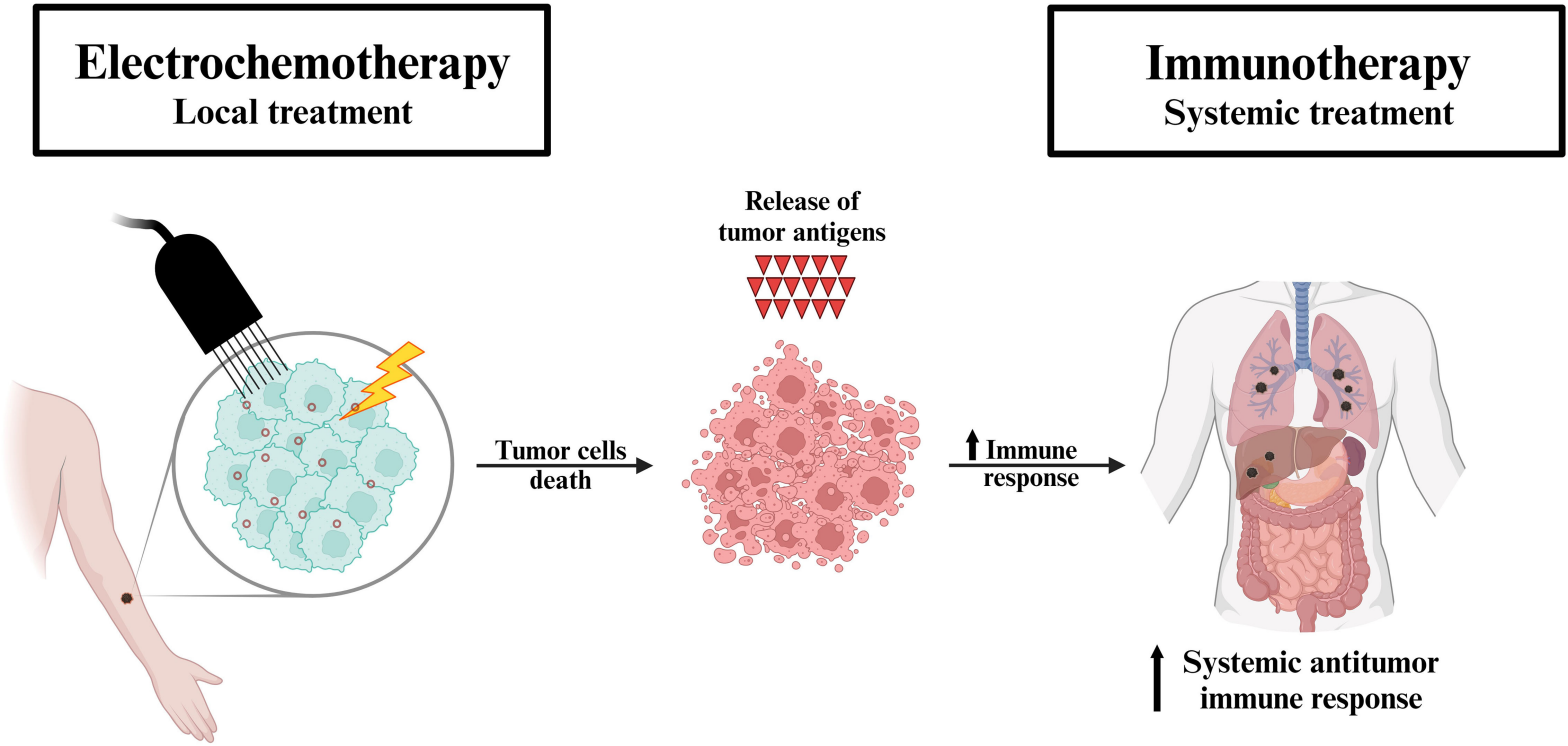
Proposed mechanism of the synergistic effect of electrochemotherapy and immunotherapy. Electrochemotherapy (local treatment) induces the immune system through immunogenic cell death (i.e. *in situ* vaccination). The tumor antigens released by the destroyed cells trigger a systemic antitumor immune response, which in turn is further exploited by immunotherapy (systemic treatment) in patients with metastatic disease.

After the initial preclinical studies, an alliance between electrochemotherapy and immunotherapy was expected. We postulated that electrochemotherapy can be considered an *in situ* vaccination inducing immunogenic cell death for adjuvant immunotherapy with ICIs (**Figure 1**) (36). By definition, *in situ* vaccination refers to any approach which exploits tumor associated antigens (TAAs) in the vicinity of the tumor to induce a TAA-specific systemic adaptive immune response (37). Specifically, the vaccination effect of electrochemotherapy was demonstrated in a study by Calvet et al. (21). In their study, CT26 murine colon cancer cells were initially successfully treated *in vitro* with electrochemotherapy with bleomycin resulting in their immunogenic cell death characterized by the release of DAMPs. Subsequently, injection of dying electrochemotherapy-treated cells into an immunocompetent mouse elicited an immune response that ultimately prevented the growth of viable cancer cells in the treated mouse. In their review article, CY Calvet and LM Mir pointed out the possible combination of electrochemotherapy with ICIs, and we have postulated that gene electrotransfer of a plasmid encoding the cytokine interleukin 12 (IL-12) may also be effective in combination with electrochemotherapy (29, 38). Both approaches were subsequently tested in several preclinical studies (34).

### 3.1 Animal studies

The combination of electrochemotherapy and IL-12 immunotherapy using gene electrotransfer has been shown to be effective in the treatment of mouse tumors. The study provided evidence that adjuvant gene electrotransfer of the IL-12 plasmid can improve tumor response to electrochemotherapy. This was particularly evident in less immunogenic tumors such as B16F10 melanoma, where the efficacy of electrochemotherapy was lower than in more immunogenic tumors, such as CT26 colon carcinoma. In contrast, in more immunogenic tumors, the efficacy of electrochemotherapy was more pronounced, and the contribution of adjuvant IL-12 therapy was less noticeable (29). In veterinary oncology, different routes of administration for IL-12 gene transfer (peritumoral vs. intratumoral) have been investigated in clinical studies combining electrochemotherapy and immunotherapy with IL-12 (39). In a recent clinical trial in canine mastocytoma tumors, we showed that the same technology, i.e. electroporation, allows simultaneous delivery of drugs and genes to the tumors and produces a better antitumor response than when electrochemotherapy and peritumoral IL-12 gene electrotransfer were subsequently administered. Tumor control and survival of dogs after combined concomitant electrochemotherapy and IL-12 gene electrotransfer were improved and prolonged, respectively (40). In addition, a study on sarcoma bearing mice showed that adjuvant intramuscular mIL-12 application after initial electrochemotherapy is dose dependent and dependent on the amount of IL-12 in the system, achieving better results in immunocompetent mice (24).

To translate this therapeutic approach to human oncology, we recently conducted a phase I clinical trial testing IL-12-encoding plasmid gene electrotransfer to basal cell carcinoma tumors. The study protocol has been published (41), the study has now been completed and the results are currently being analyzed. In further clinical trials, we intend to combine electrochemotherapy with IL-12 gene electrotransfer to investigate a possible interaction and exploitation of the *in situ* vaccination effect of electrochemotherapy. The interaction of electrochemotherapy with the ICI pembrolizumab has already been demonstrated in melanoma patients, as further described in this manuscript. The combination was not tested in the framework of a designed clinical trial with a defined treatment schedule. Electrochemotherapy was added concomitantly or adjuvant to the treatment with pembrolizumab for the treatment of cutaneous metastases (42).

## 4 Electrochemotherapy in clinical practice

Electrochemotherapy has been recognized as safe and effective treatment method for various types of cancer (7). However, in this section we will briefly discuss the clinical evidence with electrochemotherapy alone in the treatment of melanoma, breast cancer, HCC, and cutaneous squamous cell carcinoma (SCC), as the combined treatment approaches with electrochemotherapy and immunotherapy have only been published for the cancers to date.

### 4.1 Melanoma

Superficially metastatic melanoma is one of the most common indications for the treatment with electrochemotherapy (7). The first clinical studies of electrochemotherapy in the treatment of malignant melanoma were published almost 30 years ago. In 1995, our group published the first experiences with electrochemotherapy with intravenous bleomycin in melanoma patients. A complete response was achieved in 22 out of 24 treated nodules (43). Since then, many clinical studies have investigated the efficacy of electrochemotherapy in melanoma patients (44). Literature review published in 2019 included 9 case series of electrochemotherapy in the treatment of melanoma that were carried out after the publication of the ESOPE guidelines. The complete response rate ranged from 20% to 50%, while the objective response rate ranged from 60% to 100%. Electrochemotherapy had a low toxicity profile and few minor side effects, mostly in the form of local pain, erythema, and ulceration (4). Furthermore, it was shown that multiple applications of electrochemotherapy with intravenously injected bleomycin can lead to regression of even untreated skin melanoma metastases in transit. In four consecutive treatment sessions, 224 tumor nodules were treated. Although not all metastases were treated, even the untreated metastases did not progress after 9 months, suggesting an induction of locoregional or even systemic immune response (45). In addition, a recent analysis by the pan-European International Network for Sharing Practice in Electrochemotherapy (InspECT) reported an overall response rate of 82% and complete response rate of 64% in patients with melanoma. In contrast to previously published studies, they included prospectively uploaded data from 28 European centers that regularly use electrochemotherapy (46). The results of several published studies demonstrating the high efficacy, safety, and limited toxicity of electrochemotherapy have led to its inclusion in the most recent ESMO melanoma guidelines (47).

### 4.2 Breast cancer

Breast cancer is the most common neoplasm and the leading cause of cancer death in women worldwide (48). It is also the most common malignancy to metastasize to skin in women. Small single metastases can be removed surgically, while larger or multiple lesions are more challenging to treat (49, 50). For breast cancer patients who present with skin metastases, one of the treatment options is also electrochemotherapy. It is a safe and effective treatment to manage such lesions. The advantage of electrochemotherapy is that it can be performed in previously irradiated areas and can be repeated multiple times. Additionally, other systemic therapies can be concomitantly applied. The first report describing the effectiveness of electrochemotherapy in breast cancer patients was published in 1996. In a phase I/II trial, cutaneous and subcutaneous tumors were treated with electrochemotherapy. Among those patients, one metastatic breast adenocarcinoma patient, with two nodules, was also included. Both nodules showed complete responses after the treatment (51). Since then several studies have demonstrated a high response rate in providing local tumor control, with an overall response rate up to 60 - 80% and a complete response rate up to 60% (46, 52, 53).

There are various histologic types of breast cancer that differ in microscopic appearance and receptor expression, leading to differences in response to the treatment. Recently, a multicentric study investigating the efficacy of electrochemotherapy in breast cancer patients with different receptor statuses was published (54). The study demonstrated that electrochemotherapy is equally effective in the treatment of breast cancer metastases, regardless of receptor status. The response and local tumor control were better in multiple smaller lesions than in larger lesions, as previously observed. Local progression-free survival was, however, significantly lower in triple-negative type, probably due to the more aggressive cancer type and smaller choice of systemic treatments (54).

### 4.3 Hepatocellular carcinoma

A pilot study investigating the role of electrochemotherapy in the treatment of HCC was published in 2018 (55). The study included patients with unresectable HCCs located near large blood vessels where other ablative therapies are not efficient due to the heat sink effect. Ultimately, 10 patients with 17 lesions were enrolled in the study. Electrochemotherapy with bleomycin was performed in the setting of open surgery. The treatment proved to be feasible, safe, and effective, as a complete response was achieved in 88% of treated lesions (55). The promising results were confirmed in the subsequent phase II study, which enrolled 24 patients with 32 lesions. The complete response rate and partial response rate per treated lesion were 84% and 12.5%, respectively. Again, the treatment proved safe, as no major postoperative complications were observed. Only 16% of patients developed ascites due to transient liver dysfunction, which resolved spontaneously or with diuretic therapy (56).

### 4.4 Cutaneous squamous cell carcinoma

The first clinical study on electrochemotherapy in the treatment of SCC was published by Mir et al. in 1991 (57). Since then, several studies have investigated the efficacy of electrochemotherapy in the treatment of cutaneous SCC. A prospective European EURECA study of 47 patients with cutaneous SCC showed a 55% complete response rate at 2-month follow-up after electrochemotherapy. In addition, 24% of patients achieved a partial response rate, 15% had stable disease and only 4% experienced progression during treatment (58). In 2017, a retrospective study of 22 patients with cutaneous SCC treated with electrochemotherapy showed a complete response rate of 22% and a partial response rate of 59%, thus achieving a similar objective response rate to the EURECA study (59). Furthermore, a recent analysis of the InspECT registry included 162 patients with cutaneous SCC. The complete and partial response rates were 62% and 21% respectively. Better results were achieved in patients with primary and smaller (< 3 cm) tumors using intravenously administered bleomycin (60).

## 5 Combining electrochemotherapy and immunotherapy in clinical practice

Immunotherapy with ICIs has attracted substantial and broad interest since 2011, when the first ICI drug was approved by the FDA (61). Between 2015 and 2017, the number of trials with PD-1 and PD-L1 inhibitors increased from 215 to more than 1500 trials (62). A recent cross-sectional study estimated that 38.5% of cancer patients in the United States are eligible for ICI therapy (63). Electrochemotherapy is used in more than 180 centers around the world, mostly for the treatment of cutaneous tumors due to their accessibility (64). Thus, we provide an overview of cancers for which clinical reports of combined treatment with electrochemotherapy and immunotherapy with ICIs have been published. The studies are summarized in **Table 1** and explained in the following text.

**Table 1:** Clinical studies examining the combined role of electrochemotherapy and immunotherapy.

Author, year	Cancer type	Study type
Morgese et al., 2023 (65)	Melanoma	Case report
Quaresmini et al., 2021 (66)	Melanoma	Case report
Campana et al., 2021 (42)	Melanoma	Retrospective study
Karaca et al., 2018 (67)	Melanoma	Case report
Heppt et al., 2016 (68)	Melanoma	Retrospective study
Mozzilo et al., 2015 (69)	Melanoma	Retrospective study
Brizzio et al., 2015 (70)	Melanoma	Case report
Russano et al., 2021 (71)	Breast cancer	Retrospective study
Trotovsek et al., 2022 (72)	Hepatocellular carcinoma	Case report
Barca et al., 2023 (73)	Squamous cell carcinoma	Case report

### 5.1 Melanoma

The first case of a combined approach using electrochemotherapy and ICIs for melanoma was published in 2015. Initially, a patient with multiple cutaneous melanoma metastases had a partial response after 3 cycles of electrochemotherapy. Subsequently, a complete response was observed in all lesions after treatment with ipilimumab (70). Similarly, a 2016 retrospective study suggested that combined treatment with ipilimumab followed by electrochemotherapy was feasible and resulted in an increased therapeutic response of skin lesions. Thus, a local objective response was observed in 67%, while a systemic response was observed in 33% of patients with metastatic melanoma. Furthermore, T-regulatory cell counts decreased in all responders and were significantly lower than in non-responders at 3 months (69). Another retrospective study investigated the role of combined electrochemotherapy and immunotherapy with ipilimumab or PD-1 inhibitors. Authors reported objective local and systemic response rates of 67% and 22%, respectively. However, severe systemic adverse effects were observed in 25% of patients receiving ipilimumab (68). In a recent case report, a patient with metastatic melanoma progressed during the treatment with pembrolizumab with scalp metastases. These were treated with two cycles of electrochemotherapy with bleomycin, achieving partial response. Nearly complete response was eventually obtained after the treatment with ipilimumab and was present at 6-months follow-up (65). Two other case reports also demonstrated beneficial effects of combined treatment with electrochemotherapy and ICIs (66, 67).

The largest retrospective study to date investigating the combined role of electrochemotherapy and immunotherapy was published in 2021 by Campana et al. Furthermore, outcomes of patients who received pembrolizumab alone or in combination with electrochemotherapy were compared. A higher local objective response rate, longer 1-year progression-free survival, and longer overall survival in patients who received combined treatment were observed, while no serious adverse events were reported (42). In summary, several studies have demonstrated the beneficial effects of combined treatment with electrochemotherapy and ICIs for the treatment of advanced metastatic melanoma. However, these studies could not prove but could only indicate the *in situ* vaccination effect of electrochemotherapy, which enhances the systemic response to ICIs. Therefore, further translational studies are needed to investigate this effect (35). In addition, randomized controlled trials with larger numbers of patients are warranted to compare the efficacy of combined electrochemotherapy and immunotherapy with immunotherapy alone. Currently, a phase 2 multicenter, non-randomized study is enrolling patients with metastatic melanoma to determine whether concurrent treatment with pembrolizumab and electrochemotherapy is safe and can improve local and systemic response rates (ClinicalTrials.gov; ID: NCT03448666).

### 5.2 Breast cancer

To date, only one report has demonstrated the efficacy of electrochemotherapy combined with immunotherapy in breast cancer patients (71). In that study, 55 metastatic breast cancer patients were treated with electrochemotherapy 78 months after the primary breast cancer diagnosis (range 14–441 months). At the time of electrochemotherapy, 27% of patients did not receive any concomitant therapy, 27% received chemotherapy, 25% hormonal therapy, 5% immunotherapy, and 14% combined therapy (5 chemotherapy + immunotherapy, 5% hormonal therapy + immunotherapy). Overall, a complete response rate was observed in 64% of patients, while 22% of patients had a partial response and 14% had stable disease. Regarding the concomitant treatment, the efficacy of electrochemotherapy combined with immunotherapy was almost 100%, while when electrochemotherapy was combined with chemotherapy the complete response was lower (67%). The progression-free survival and overall survival at 24 months were higher in patients who were treated with electrochemotherapy combined with immunotherapy compared to other treatments. Unfortunately, at 36 months this benefit was no longer observed. The study showed the efficacy of electrochemotherapy combined with immunotherapy in providing a local tumor control rate, as well as short-term progression-free and overall survival benefits. However, it should be noted that only three patients in this study received combined treatment with electrochemotherapy and immunotherapy, therefore larger studies are needed to examine the combined treatment in breast cancer patients (71).

### 5.3 Hepatocellular carcinoma

To our knowledge, only one case of combined treatment with electrochemotherapy and immunotherapy in a patient with HCC has been published (72). A 43-year-old patient with multifocal HCC in both lobes of the liver and cirrhosis was initially treated with 24 cycles of bevacizumab and atezolizumab. Subsequently, electrochemotherapy with bleomycin was used for the remaining two lesions in segment 3 of the liver, and a complete response was observed 3 months after treatment. However, because the patient had only two lesions at the time of electrochemotherapy and the follow-up period was relatively short, the synergistic role of both treatments could not be fully determined (72). Therefore, further studies are needed to investigate the combined role of electrochemotherapy and immunotherapy for HCC (74).

### 5.4 Cutaneous squamous cell carcinoma

Recently, Barca et al. have published a case of combined treatment of electrochemotherapy and immunotherapy in a patient with advance-stage cutaneous SCC (73). An 80-year-old patient with a large, bleeding, frontotemporal cutaneous SCC was initially treated with two cycles of electrochemotherapy. As early disease progression was detected, the patient underwent experimental treatment with 24 cycles of cemiplimab followed by a further two cycles of electrochemotherapy. Eventually, remission of the disease on the skin side was demonstrated with multiple incisional biopsies. Unfortunately, the disease progressed to the orbital cavity and patient died after four months of combined treatment with electrochemotherapy and immunotherapy. The authors concluded that the combined treatment was effective in both controlling disease progression and alleviating patient’s symptoms.

## 6 Conclusions

Electrochemotherapy is a highly effective, locoregional, ablative therapy that is predominantly used for the treatment of cutaneous tumors and, more recently, for the treatment of deep-seated tumors. Electric pulses applied to the target lesion temporarily increase the permeability of the cell membrane and subsequently enhance the delivery of low-permeant cytotoxic drugs to the tumor cells, leading to their death. In addition, electrochemotherapy could be considered an *in situ* vaccination, as it induces an immune response through immunogenic cell death, which in turn can trigger the efficacy of immunotherapy. The clinical evidence for combined treatment with electrochemotherapy and immunotherapy is still lacking. To date, only a few retrospective studies on patients with melanoma and breast cancer and a case reports on a patient with HCC and cutaneous SCC have shown a beneficial role of the combined treatment in the short term.

In summary, combined treatment with electrochemotherapy and immunotherapy could improve local tumor control and boost the systemic antitumor response. Moreover, electrochemotherapy as a form of *in situ* vaccination could also broaden the applicability of immunotherapy in different cancer types. Nevertheless, further randomized controlled trials with a larger number of patients and a longer follow-up period are warranted to better investigate the (positive) role of the combined treatment. Furthermore, additional preclinical and translational studies are needed to better explain the underlying mechanism of the combined treatment.

## Author contributions

BH: Conceptualization, Writing – original draft. MO: Writing – original draft. BT: Writing – review & editing. MC: Conceptualization, Supervision, Writing – review & editing. TJ: Writing – review & editing. GS: Conceptualization, Supervision, Writing – original draft, Writing – review & editing. MD: Supervision, Writing – review & editing.
